# Understanding the Dynamics of Cellulose Dissolved in an Ionic Liquid Solvent Under Shear and Extensional Flows

**DOI:** 10.1021/acs.biomac.1c01623

**Published:** 2022-04-20

**Authors:** Crystal E. Owens, Jianyi Du, Pablo B. Sánchez

**Affiliations:** † Hatsopoulos Microfluids Laboratory, Department of Mechanical Engineering, Massachusetts Institute of Technology, Cambridge, Massachusetts 02139, United States; ‡ Applied Physics Department, Experimental Science Building,Universidade de Vigo, 36310 Vigo, Spain

## Abstract

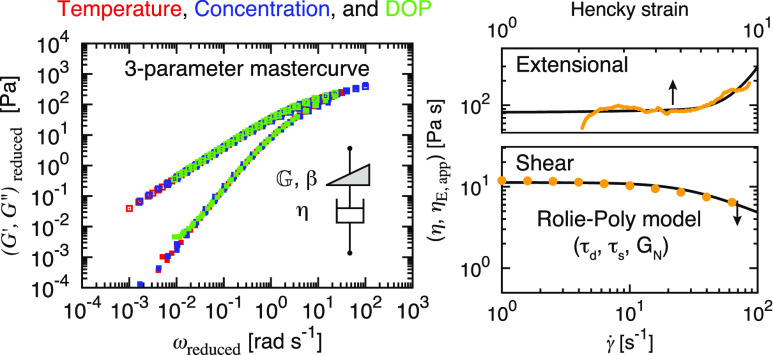

Ionic liquids (ILs) hold great potential as solvents to dissolve, recycle, and regenerate cellulosic fabrics, but the dissolved cellulose material system requires greater study in conditions relevant to fiber spinning processes, especially characterization of nonlinear shear and extensional flows. To address this gap, we aimed to disentangle the effects of the temperature, cellulose concentration, and degree of polymerization (DOP) on the shear and extensional flows of cellulose dissolved in an IL. We have studied the behavior of cellulose from two sources, fabric and filter paper, dissolved in 1-ethyl-3-methylimidazolium acetate ([C_2_C_1_Im][OAc]) over a range of temperatures (25 to 80 °C) and concentrations (up to 4%) that cover both semidilute and entangled regimes. The linear viscoelastic (LVE) response was measured using small-amplitude oscillatory shear techniques, and the results were unified by reducing the temperature, concentration, and DOP onto a single master curve using time superposition techniques. The shear rheological data were further fitted to a fractional Maxwell liquid (FML) model and were found to satisfy the Cox–Merz rule within the measurement range. Meanwhile, the material response in the non-LVE (NLVE) regime at large strains and strain rates has special relevance for spinning processes. We quantified the NLVE behavior using steady shear flow tests alongside uniaxial extension using a customized capillary breakup extensional rheometer. The results for both shear and extensional NLVE responses were described by the Rolie-Poly model to account for flow-dependent relaxation times and nonmonotonic viscosity evolution with strain rates in an extensional flow, which primarily arise from complex polymer interactions at high concentrations. The physically interpretable model fitting parameters were further compared to describe differences in material response to different flow types at varying temperatures, concentrations, and DOP. Finally, the fitting parameters from the FML and Rolie-Poly models were connected under the same superposition framework to provide a comprehensive description within the wide measured parameter window for the flow and handling of cellulose in [C_2_C_1_Im][OAc] in both linear and nonlinear regimes.

## Introduction

With rising environmental concerns worldwide,^1^ the transition from a linear toward a circular economy has gained the attention of industry, institutions, and the public opinions.^2^ A sustainable approach should drive toward an efficient use of natural resources, which is fostered by the use of materials with a lower environmental impact and higher recyclability.^3^ Cellulose is among the key materials building momentum in the industrial transition to come due to its widespread availability, durability, and renewable and biodegradable properties as a natural fiber. The chemical structure of cellulose, in which glucose rings linked by β-1,4 glycosidic bonds confer high chemical and thermal stability,^4^ makes it difficult to process without degrading the polymer chains, an effect magnified for cellulose with a high degree of polymerization (DOP). Meanwhile, these chains are linked via intra- and interchain hydrogen bonds (HBs),^5^ which create an amphiphilic biopolymer with tightly packed, crystalline subdomains whose dissolution happens very rarely under mild conditions. *N*-Methylmorpholine-*N*-oxide (NMMO) and^6^ copper complexes (e.g., CuEN) in aqueous media^7^ or the solutions of lithium chloride in *N*,*N*-dimethylacetamide (LiCl/DMAc)^8^ are among the few solvents that can disrupt HB networks without a massive destruction of glycosidic bonds, which would otherwise result in a significant reduction of the DOP due to dissolution alone. Nonetheless, apart from NMMO, the application of these solvents on an industrial scale has been limited for economic, health, and safety reasons, and by the environmental impacts of handling of these solvents, and the scale of use of NMMO remains limited compared to viscose-like products. More recently,^9^ ionic liquids (ILs) have demonstrated comparable effectiveness in dissolving cellulose at a molecular level with relatively little damage to the polymer chains. Since then, remarkable advances have been made in the processing of cellulose in ILs.^10^

ILs, originally defined as salts melting under 100 °C,^11^ consist of large ions with multiple conformations,^12^ leading to solvents with extremely low volatilities,^13^ while remaining liquid over a wide range of temperatures.^14^ Given the large number of ions and ion combinations, the properties of an IL can be tuned by a suitable selection of its constituents. Hence, these are known as “designer solvents”. Good candidates to dissolve cellulose are expected to disrupt HB networks effectively.^15^ This capability is sometimes quantified by the Kamlet–Taft parameter,^16^ which is defined by the strength of an anion to act as an HB acceptor. This is a common ability for acetate-, phosphate-, or chloride-based ILs. Although the role of the cation remains an open question, previous work has mostly adopted imidazolium derivatives.^17,18^ More recently, a family of ILs described as “superbases”^19^ has also been applied with promising results. Numerous investigations have been conducted to elucidate the thermodynamics of dissolution from the perspectives of the solvent^20−25^ and cellulose.^8,26^ Dissolution kinetics are strongly dependent on the viscosity of the solvent media,^27^ which increases sharply as the cellulose dissolves and with temperature. Given that pure ILs are Newtonian liquids whose viscosities are already high, and the values generally vary from 100 to 500 mPa s at 25 °C, the slow kinetics of cellulose dissolution constrains the practicable solubility. To overcome this limitation, the use of nonprotic polar cosolvents has been shown to help dissolution speed,^28−30^ despite the increasing difficulty in recycling the solvent and cosolvent.

Once the cellulose dissolves, the liquid solution is often referred to as the “dope”. Results from small angle X-ray scattering indicate that cellulose in alklyl imidazolium acetate solvents is generally molecularly dissolved^31,32^ even when present at very high concentrations,^33^ and scattering profiles fit the model of a cylinder with a cellulose core surrounded by a diffuse ordered shell made of the IL solvent molecules.^32^ In some IL mixtures such as aqueous tetrabutylammonium hydroxide, results from small angle X-ray scattering and light scattering suggest that the aggregation of the cellulose may be present even at dilute concentrations.^34^

To finally obtain regenerated cotton fibers^35−37^ or polymer composites,^38,39^ more complex fluid handling is required through mixing apparatuses, nozzles, or other piping, and researchers must obtain an intimate understanding of the shear and extensional rheology of the cellulose solutions to fully understand how the cellulose behaves (which often involves polymer degradation) during the handling. Previous studies have identified key parameters to include cellulose DOP,^40−43^ solvent viscosity,^44^ solvent quality^44,45^ (to quantify cellulose–solvent interaction), concentration,^45−48^ and temperature.^46^ For economic reasons, a high concentration of cellulose is preferred, which drives most “spinnable” dopes into entangled regimes.^10,49^ As a result, a moderately high temperature is required to prevent high viscosity from excessively slowing the dissolution of cellulose and to facilitate the processing of cellulose solutions, especially at a scaled-up level. Consequently, the comprehensive effect of temperature and concentration on the structure of the polymer dissolution under stress should be understood in detail for an optimal process design. In addition, the dynamics of polymer solutions rely heavily on the molecular weight of the polymer chains,^43,50,51^ often referred to in terms of DOP for cellulose chains. A cellulose chain ranges from a few hundreds to several thousands of monomeric units (glucose rings). Significant chain breaking has been reported when cellulose and ILs coexist for several hours at a temperature above 90 °C.^40^

The knowledge of cellulose spinning relies on empirical tests and heuristic rules,^52,53^ among which it is assumed that long polymer chains help with the fiber formation due to their extensibility.

Thus, the effect of DOP on shear and extensional flows is highly relevant to fiber spinning. On the solvent side, moderately high viscosities that decay exponentially with temperature inverse according to the Arrhenius relation^54^ should be expected. Also, the solvent quality incorporated by the Mark–Houwink–Sakurada (MHS) equation^49^ plays a relevant role when analyzing the rheology of polymer mixtures to understand the dependence of viscosity on cellulose concentration. The MHS equation relates intrinsic viscosity with its DOP and establishes a qualitative criterion which accounts for the interactions between polymers and solvents.^51^

**Figure 1:**
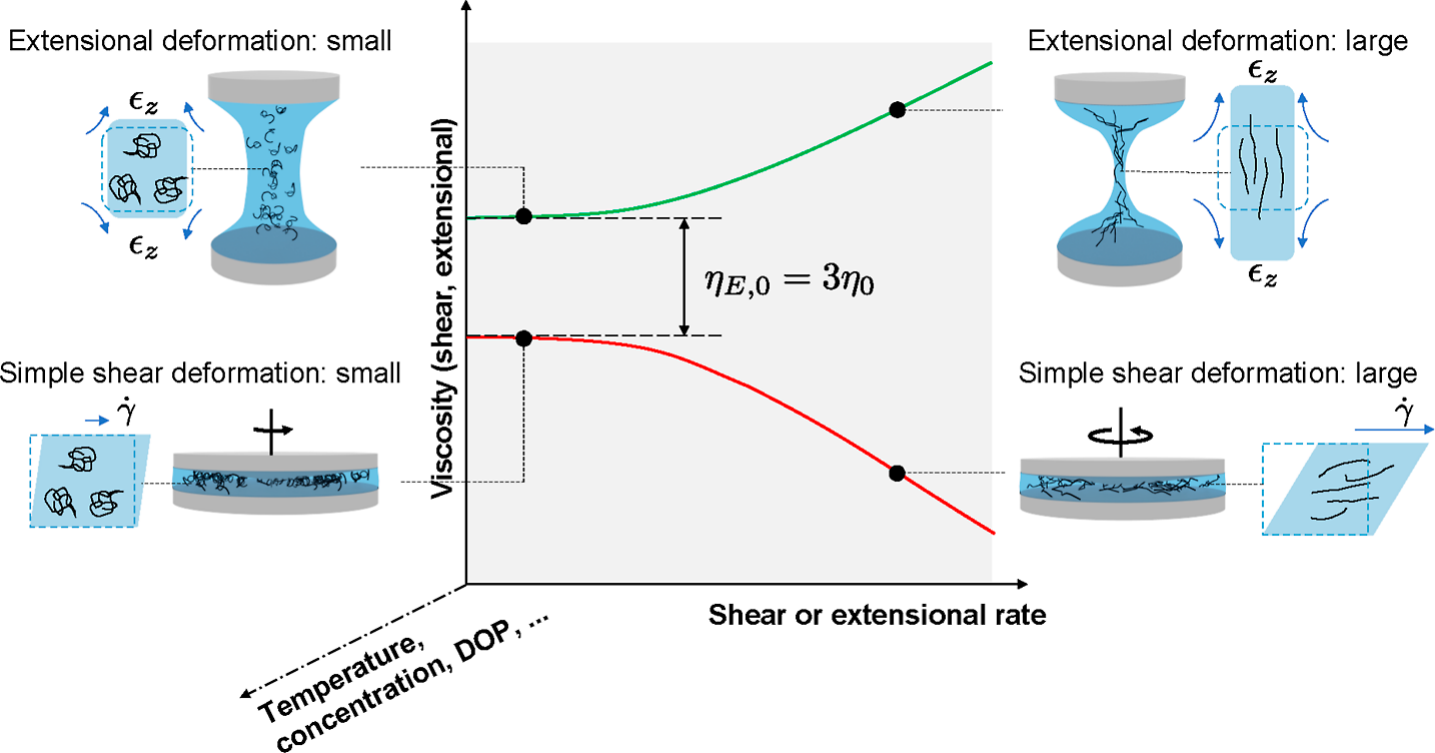
Overview of the polymer deformation and structure–rheology relationships in shear and extensional flows. The zero-rate properties in both flows are connected by a Trouton ratio of 3, and their trends diverge as the strain rate increases, corresponding to distinct material responses to different flow types at large deformation.

The dynamics of cellulose/IL solutions have primarily been characterized by shear rheology,^15^ in which the liquid is confined and mechanically deformed in a simple shear flow, as depicted in Figure 1. By probing the viscoelastic response, the resulting properties have been related to material parameters such as the DOP, concentration of cellulose, and temperature. These parameters have been further used to measure the solvent quality of the IL.^15,55^ In a steady shear flow, a transition from Newtonian to shear-thinning behavior can be observed when the concentration is sufficiently high.^15^ In addition, multiple cellulose/IL combinations have been shown to violate the Cox–Merz rule. This deviation happens at high shear rates and angular frequencies, and it has been attributed to associative networks forming between cellulose chains.^15,45^ The rheological characterizations as shown in shear flows are most productively generalized by the application of time–temperature superposition (tTS), in which tests at low temperatures can be equivalently shifted to higher frequencies at standard temperatures and vice versa.^56,57^ This technique allows for measurements at a wide range of oscillatory frequencies to probe the rubbery plateau region, which is unattainable by direct measurements within the capability of most shear rheometers. This technique has been extensively used to quantify the distinct timescales in a cellulose/IL solution.^45^ Recently, an analogue of tTS named time–concentration superposition (tCS) has been established, which applies the counter-influence of measured time and concentration below the entanglement concentration in a similar manner.^48,58^

Furthermore, shear rheology has also been used to investigate the coagulation process upon the addition of water to the cellulose/IL solution, which leads to a better understanding of the effects of solution rheology on the spinning process.^53,59^ These results have shown that the elastic modulus increases linearly with the amount of water up to a saturation point, and the fracture energy of these hydrated cellulose/IL samples has been linked to the stability of a spin line. Additional work with unhydrated cellulose solutions failed to show a clear connection between shear rheology and spinnability by dry-jet or wet solvent spinning methods. However, solution viscosity has been shown to remain a driving role in the spinnability of cellulose solutions via electrospinning.^58^ The more complex rheology of cellulose solutions has not been well defined, and consequently, it has not been applied to understand fluid behavior under intricate processing control.

As these results may indicate, shear rheology is a useful descriptor but does not comprehensively reflect how cellulose/IL solutions will behave under nonlinear deformation in more complex applications. Instead, a more critical mode of deformation is uniaxial stretching, as depicted in Figure 1, in which a cylindrical liquid element is uniformly extended. This type of flow depicts a highly nonlinear directional deformation, and variations in the polymer conformation of cellulose play a fundamentally different role from that in shear flows.^48,60^ The fluid properties exhibited in uniaxial stretching are closely connected to the fiber spinning processes.^61^ For example, the extensional-thickening behavior is a critical parameter for forming a stable spin line and can be directly characterized in an extensional flow.^60^

In this paper, we investigate both the shear and extensional rheology of cellulose/IL solutions at varying temperatures, concentrations, and DOP, and subsume the results into a general description of the rheological behavior at varying material conditions, as shown in Figure 1. We begin with a brief theoretical overview of the criteria for selecting appropriate constitutive models. Experimentally, we analyze the counteracting effects of the temperature, concentration, and DOP on the shear and extensional rheology of cellulose dissolved in 1-ethyl-3-methylimidazolium acetate ([C_2_C_1_Im][OAc]). Moderate ranges of the concentration (0.5 to 4%) and temperature (25 to 80 °C) are selected, which approximate the processing window for a real spinning process to minimize cellulose degradation. Subsequently, linear viscoelastic (LVE) regime rheology was studied via small-amplitude oscillatory shear (SAOS). The fractional Maxwell liquid (FML) model was used to describe the master curves and the obtained constitutive parameters are compared and discussed. We generalize the rheological behavior of the measured solutions from tTS and tCS, and by a new shifting technique using the cellulose DOP, namely time–DOP superposition (tDS). Furthermore, the nonlinear regime was studied under transient extensional flows using a customized capillary breakup extensional rheometer (CaBER). Although shear-thinning behavior is identified as expected, the rheological behavior in an extensional flow becomes increasingly complex with a nonmonotonic trend in the evolution of extensional viscosity. By applying a constitutive model based on the reptation theory for solutions in the entangled regimes, we render a unified set of rheological parameters to accurately describe the complex fluid behavior in both shear and extensional flows. The extracted model parameters are further correlated with the cellulose structure in different flow scenarios. Finally, the relationship between the flow and the structure of the polymer was integrated and discussed in the context of the ultimate processing of cellulosic fibers.

## Materials and Theoretical Backgrounds

### Materials and Methods

Two cellulose sources, cotton fibers from textiles (DOP = 2710; Inditex S.A.) and filter paper (DOP = 1340; Whatman plc), were dissolved in 3-ethyl-1-methylimidazolium, [C_2_C_1_Im][OAc] (purity of 90%; Sigma-Aldrich, CAS number 143314-17-4). All the products were used without further purification. Mixtures were weighed with precision to ±1 × 10^–4^ and placed into sealed vials. Cellulose was dissolved under gentle (≈1 Hz) magnetic stirring at 80 °C. Dissolution time varies from 12 to 36 h depending on the cellulose source and the concentration. Although these conditions were required to ensure full dissolution in a reasonable timeframe, this preparation is known to result in a mild degradation of cellulose chains over the time of dissolution (see the Supporting Information for details).^40,42,62^ Once the cellulose was fully dissolved, the fluids were removed from the hot plate and stored at room temperature.

The shear rheology is characterized on a Discovery Hybrid Rheometer 3 (TA Instruments) equipped with a parallel-plate geometry (40 mm in diameter, with a geometry gap of 500 μm) or a cone-and-plate geometry (40 mm in diameter, with a cone angle of 2°, and a truncation gap of 55 μm), both made with aluminum. To minimize the exposure time of fluids to ambient humidity, a solvent trap was consistently applied during tests with mineral oil (CAS 8042-47-5; Sigma-Aldrich) as the trap fluid. The rheometer uses a Peltier heating system on the lower plate to precisely control the temperature of the equipped geometry set. Shift factors in temperature superposition were calculated using TRIOS software (TA Instruments).

The extensional rheology is measured using a customized CaBER.^63^ In this device, a sample volume is placed between two coaxial disks and is subsequently separated in a step-strain manner. The resulting filament formed between two liquid reservoirs undergoes a capillarity-driven self-thinning process, and their filament thinning dynamics, monitored through the temporal evolution of the filament diameter, can be used to extract the extensional rheological properties such as the extensional viscosity and the longest relaxation time.^64^ In this study, a disk diameter of 6 mm was selected with a step Hencky strain of 1.35. The temporal evolution of the filament diameter is monitored using a commercially available high-speed camera (Phantom M320 s, Vision Research Inc.) at 2000 frames per second at a resolution of 17 μm/pixel.

### Time Superposition

The SAOS results were reduced using time superposition techniques, from which the storage and loss moduli (*G*′ and *G*″) at different experimental conditions were collapsed onto a master curve.^65^ Here, we applied this method to reduce multiple variables using tTS, tCS, and tDS. In each superposition scenario, the system with the highest viscosity was chosen to be the reference. Shifting parameters were calculated using TRIOS software as shown in eq 1 as
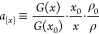
1where the modulus *G* is either *G*′ or *G*″, the storage or loss modulus, and [*x*] denotes the superimposing variable (temperature in Kelvin, concentration, and DOP in tTS, tCS, and tDS, respectively). The material density ρ varies slightly within the measurement range of the temperature and concentration, that is, ρ(*T* = *T*_0_)/ρ = ρ_0_/ρ ≈ 1, where *T*_0_ is the reference temperature. When superimposing with temperature, the Arrhenius relation arises as described in eq 2, in which the activation energy of the flow, Δ*H*, can be obtained from the horizontal shifting as
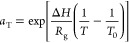
2where *R*_g_ ≈ 8.314 J/(K mol) is the universal gas constant. The vertical shifting follows the convention of the Rouse model as^66^
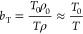
3in which the previous isopycnic assumption on density is applied.

### Linear Viscoelasticity

To describe the LVE behavior over a wide relaxation spectrum, the superimposed master curves can be fitted into the fractional Maxwell model (FMM) with the single-mode relaxation modulus expressed as^67^
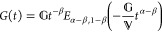
4in which the Mittag-Leffler function *E*_*a*,*b*_(*z*) is expressed as^67^
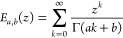
5where Γ(*ak* + *b*) is the Gamma function.

The two parameters  and  are referred to as quasi-properties and their units are determined by the fractional exponents α and β, respectively.^68^ When α is set to unity, the FMM is reduced to the FML model to describe a solvent contribution that exhibits Newtonian fluid behavior, and the quasi-property  is reduced to the dimension of viscosity. The dynamic moduli (*G*′ and *G*″) can be readily obtained by taking the Fourier transforms of eq 4 and are expressed in equation as
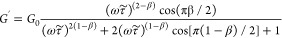
6a
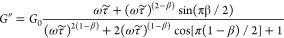
6bwhere the front factor  and the timescale , both derived from dimensional analysis, describe the magnitudes of both moduli and the dominant relaxation time, respectively. From asymptotic analysis, both storage and loss moduli exhibit distinct scaling laws at low and high frequencies. When ωτ̃ ≪ 1, both moduli scale as *G*′ ∼ ω^2−β^ and *G*″ ∼ ω, while as ωτ̃ ≫ 1, both *G*′ and *G*″ scale with ω^β^ with different front factors^69^ (see the Supporting Information).

### Tube Models

As the strain rate increases, the advection of flow drives a structural change to the polymer conformation beyond its linearly extensible regime, resulting in nonlinear rheological behavior for the bulk spinning dope. Previous studies have used constitutive models for dilute or semidilute polymer solutions, such as the FENE-P model^70^ or the Giesekus model,^71^ to describe this nonlinear variation. However, in these models, the polymer–polymer interactions at high concentrations well above the overlap concentration ([cel] ≫ [cel]*) are not fully accounted for or are predominantly phenomenological, thus the discrepancy in rheological behavior between shear and extensional flows cannot be accurately interpreted. In this study, we apply tube models to render a more comprehensive description of semidilute and concentrated cellulose solutions while retaining physical interpretations for the constitutive parameters. In the tube model, a polymer chain is confined by its neighboring polymers in a mean-field tube.^72^ These tubes interact with each other at their topological crossovers, or entanglements. Within these tubes, polymer chains are inhibited for moving transversely and can only reptate along the tube primitive length at a much decreased diffusivity, thus a significant slowdown in relaxation.^66,73^ Based on this idea, Doi and Edwards proposed the first full-dimensional constitutive equation of the tube model.^74^ In their model, tubes are reoriented based on the independent alignment approximation. As a result, the stress is solely expressed by the affine tube rotation. Despite its success in describing the behavior of a series of low-density monodisperse linear polymers, the Doi–Edwards model is well-known for predicting excessive rate thinning at high strain rates that can lead to flow instabilities.^66^ Nevertheless, this pioneering work has inspired a series of subsequent models that incorporate additional mechanisms to describe increased rheological complexity, such as the Doi–Edwards–Marrucci–Grizzuti model^75,76^ adding the stretch of polymer chains, the Graham–Likhtman-and-Milner–McLeish model adding the convective constraint release (CCR) effects,^77^ the following double constraint release with chain stretch model^78^ and the rouse linear entangled polymers (Rolie-Poly) model^79^ that combines both the contour length fluctuation and the CCR effects. Because of the model versatility and for mathematical simplicity, we apply the Rolie-Poly model with infinite polymer chain extensibility to describe the nonlinear rheological behavior of cellulose solutions in both shear and extensional flows over the measurable range.

In the Rolie-Poly model, the constitutive equation is expressed in the form of a self-evolving conformation tensor ***C***, which evolves according to eq 7a as
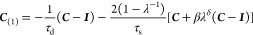
7a
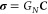
7b

In this equation, two timescales, τ_d_ and τ_s_, arise as the disengagement time and the Rouse time to characterize the tube reorientation and polymer chain stretch, respectively. The plateau modulus *G*_*N*_ describes the magnitude of stress due to tube alignment. The subscript “(1)” describes the first-order upper-convected derivative.^65^ Two dimensionless parameters β and δ describe the magnitude of the CCR effect. In this study, a set of values with β = 1 and δ = −0.5 is adopted that produces analytical predictions that are consistent with molecular theories.^79−81^ The polymer stretch is defined as . A number of previous studies^60,66,82^ have featured distinct relaxation times manifested in shear and extensional flows due to the complex variations of polymer chain conformation induced by tube reorientation and polymer chain stretch. Compared with previous studies on dilute or semidilute polymer solutions with a single relaxation timescale, the application of tube models allows for more comprehensive characterizations of rheological complexity for polymer solutions in the entangled regime.

### Extensional Rheology

The extensional rheology of cellulose/IL solutions is quantified using capillarity-driven thinning techniques. A liquid filament undergoes the self-thinning process driven by the capillary pressure and resisted by the viscoelastic responses of the tested material. In the absence of inertial effects (taking the Ohnesorge number ) and gravitational effects (taking the Bond number Bo ≡ ρ*gR*^2^/Γ ≪ 1), we can write the stress balance equation as^83^
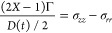
8where Γ is the surface tension, and *D*(*t*) is the temporally evolving filament diameter. The geometric correction factor *X* quantifies the deviation of a filament shape from being cylindrical, and its value can be both time and model dependent as the filament shape evolves.^84^ For a Newtonian fluid under visco-capillary balance, this value is constant at *X* = 0.7127.^83,85^ In this study, a value of Γ = 47 mN/m^48^ was adopted from the pure IL, and variations in this value due to the addition of cellulose did not affect the overall viscosity trend. From eq 8, the extensional viscosity can be calculated as
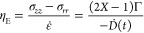
9where the filament strain rate can be calculated as ε̇ = −2*Ḋ*(*t*)/*D*(*t*).

In this study, the rheometric data from shear and extensional flows are fitted to both the FML and the Rolie-Poly model. A unified set of constitutive parameters can be obtained regardless of the flow type, and a comparison of material responses can be made between shear and extensional flows.

## Results and Discussion

**Figure 2:**
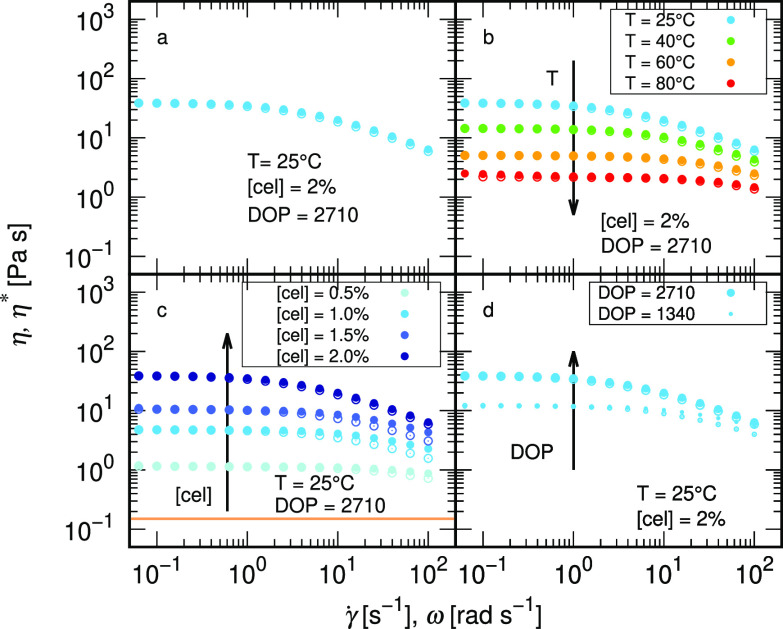
Shear viscosity (η(γ*˙*), filled symbols) and complex viscosity (η*(ω), hollow symbols) as a function of shear rate and angular frequency, respectively, for cellulose dissolved in [C_2_C_1_Im][OAc]. (a) Shear and oscillatoray data for [cel] = 2% and DOP = 2710 at 25 °C. Other conditions are fixed as noted, systematically varying only (b) temperature and (c) concentration, where solid horizontal line is the viscosity of the solvent or (d) DOP.

In Figure 2, we show the dependence of steady shear viscosity η(γ*˙*) (filled symbols) and complex viscosity η*(ω) (hollow symbols) as a function of the shear rate and angular frequency, respectively. Figure 2a shows the results with a cellulose DOP of 2710, a weight concentration of 2%, and a temperature of 25 °C, which is subsequently taken as a benchmark for the comparison of materials under different conditions. The results show good agreement with the Cox–Merz rule within the measurement range, evidenced by the general agreement observed between steady shear and complex viscosities. As temperature varies from 25 to 80 °C (Figure 2b), the viscosity decreases over 2 orders of magnitude, and shear-thinning behavior is initiated at lower rates, while the Cox–Merz rule continues to hold. These observations are consistent with the previous reports for cellulose in acetate^46,51^ and chloride ILs.^86,87^

In contrast, increasing concentration has the opposite influence on the overall rheology, as shown in Figure 2c. In this figure, the dope viscosity grows orders of magnitude with the addition of polymers, even at low concentrations. Note that [C_2_C_1_Im][OAc] is a Newtonian fluid with a viscosity of approximately 0.150 Pa s at 25 °C (see the Supporting Information). The effect of cellulose concentration on the rheology of cellulose in [C_2_C_1_Im][OAc]^46,48,51^ applies to other IL solutions as well.^45−47,50,58,86^ Regardless of the solvent quality, higher concentrations lead to slower dynamics and more significant shear-thinning phenomena. Notably, we capture this trend at accessible shear rates because of the high DOP of our cellulose; typically, the shear rate required to see this well would be much higher so as to be inaccessible for cellulose with a lower DOP.^46,88,89^ The effect of the cellulose DOP or the length of cellulose chains—on the rheological behavior is relatively unstudied due to a lack of high-DOP cellulose sources and finds less focus in the standing literature.

In Figure 2d, two cellulose sources with a DOP of 1340 and 2710 are compared. The monomer in cellulose is a glucobiose unit with a molecular weight of 162 Da, so these correspond to overall polymer weights of 217.1 and 439.2 kDa, respectively. The results are consistent with the trends shown in previous studies,^40,43,58^ revealing that under the same experimental conditions, longer cellulose chains lead to slower dynamics and more evident shear-thinning behavior, even at lower shear rates.

Polymer dissolution in entangled/concentrated regimes is likely to show deviations from the Cox–Merz rule due to nonaffine conformation and the disengagement of polymer chains in a steady flow, leading to a lower shear viscosity at high rates. Quite notably, reversed effects have been reported^15^ for cellulose/IL solutions. Although this has not yet been explained, Chen et al.^45^ have attributed this discrepancy to polymer association/clusters leading to time-dependent “cross-linking” associated with HBs between chain-anion-chain. Therefore, steady shear flows can promote interchain bonding more than oscillatory shear. The overall process delays chain breakup to a higher shear rate, as has been described for known associative polymer systems.^90^ The results presented in this work (see Figure 2 and Supporting Information) broadly follow the Cox–Merz rule within the probed range of the shear rate, with some minor deviations for intermediate cellulose concentrations.

Shear thinning at low shear rates is sometimes reported for cellulose in other ILs, which may indicate aggregation of the cellulose,^34,50^ although part of the reported signal may be due to measurements close to the instrument noise floor or to interfacial effects, which are known to influence rheological measurements of low-concentration cel/IL.^91^ In [C_2_C_1_Im][OAc], X-ray scattering studies of cellulose at a range of DOP and concentrations covering the range of those in our study unambiguously indicate molecular-level dissolution without significant aggregation.^31−33^ The solution structure has been found to fit a coaxial double cylinder model with a core made of individual cellulose chains and a diffuse shell made of the IL solvent molecules.^33^ This is reflected in our rheological measurements by a lack of shear thinning at low shear rates, indicating a lack of low-level aggregates, and by adherence to the Cox–Merz rule at high shear rates, which suggests that cellulose behaves here as a polymer chain in [C_2_C_1_Im][OAc] without extraneous interchain interactions. At much higher frequencies (i.e., within the entanglement regime), we would no longer expect the Cox–Merz relationship to hold due to the demonstrated associativity of entangled cellulose systems.^45^ We further observe a temperature-independent intrinsic viscosity in our solutions, suggesting a lack of substantial interfacial activity in our rheometer tests (see the Supporting Information).

**Figure 3:**
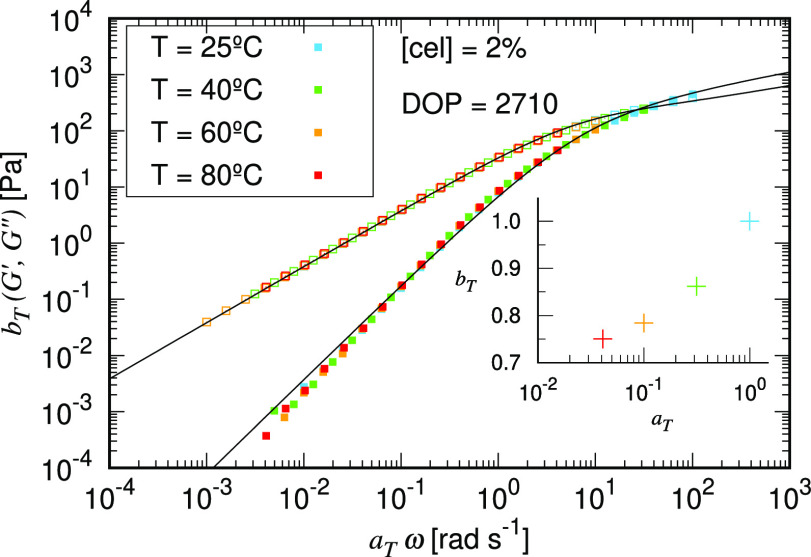
SAOS master curve obtained via tTS for cotton fibers (DOP = 2710) dissolved in [C_2_C_1_Im][OAc] at 2% cellulose concentration, referenced at 25 °C, with solid symbols indicating *G*′ and hollow symbols indicating *G*″. The solid lines represent the fitting to the FML model. The shifting factors *b*_T_ and *a*_T_ are shown in the inset plot.

**Figure 4:**
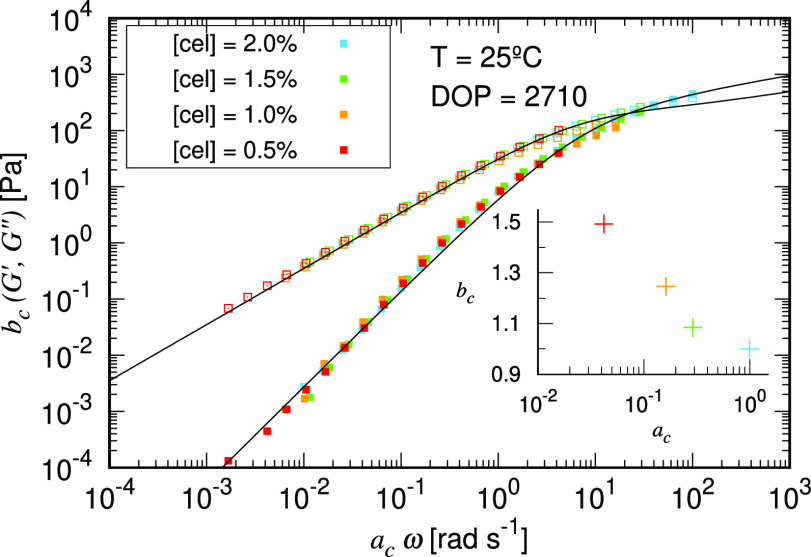
SAOS master curve obtained via tCS for cotton fibers dissolved in [C_2_C_1_Im][OAc] at 25 °C, with solid symbols indicating *G*′ and hollow symbols indicating *G*″. The solid line represents the same fitting to the FML model as in Figure 3. The shifting factors *b*_C_ and *a*_C_ are shown in the inset plot.

The linear viscoelasticity at different temperatures and concentrations for both cellulose sources (DOP = 2710 and 1340) is investigated through SAOS measurements. As shown in Figure 3, the dynamic moduli collapse onto a single master curve upon the application of tTS, which indicates the absence of phase transitions or other irreversible structural variations during the oscillatory shear. The collapsed data of storage and loss moduli are fitted with the FML model (black solid lines) simultaneously. Extracted vertical (*b*_T_) and horizontal (*a*_T_) shifting parameters are plotted in the inset of Figure 3. Because elastic modulus measurements become noisy at low concentrations and at high temperatures, the frequency range is shortened accordingly. We further fitted *a*_T_ with an Arrhenius relation equation to obtain the activation energy for the flow. The results, depicted in Supporting Information, show a monotonic increase with cellulose concentrations at fixed DOP. A similar trend is captured at a fixed concentration (2%) when the DOP increases. Both patterns are consistent with the previous work involving different ILs.^43,46,51^ Similarly, tCS is applied to the fiber solutions at *T* = 25 °*C*, and the results collapse consistently, as shown in Figure 4 with similar fitting to the FML model as in Figure 3. The shift factors *a*_C_ and *b*_C_ (in the inset of Figure 4) fall into the same numerical range as the temperature shifting factors, *a*_T_ and *b*_T_, now with *b*_C_ showing an increasing trend with concentration. Moreover, the fitted activation energies from our rheometric shift factors find quantitative agreement with activation energies extracted from ^1^H NMR measurements on microcrystalline cellulose/[C_2_C_1_Im][OAc] at varied concentrations,^92^ indicating that slower diffusion of the solvent ions due to greater ion–cellulose association is one contribution to the decreasing shift factor (increasing activation energy) with increasing concentration.

From Figures 3 and 4, the time superposition law is generic to different variables. Naturally, we seek a general superposition law to comprehensively collapse the experimental data at varying temperatures, concentrations, and DOP onto a single master curve. Mathematically, this general superposition law corresponds to a generalized horizontal shifting factor *a*(*T*, [cel], DOP) that applies to the variation of any single or multiple variables.

**Figure 5:**
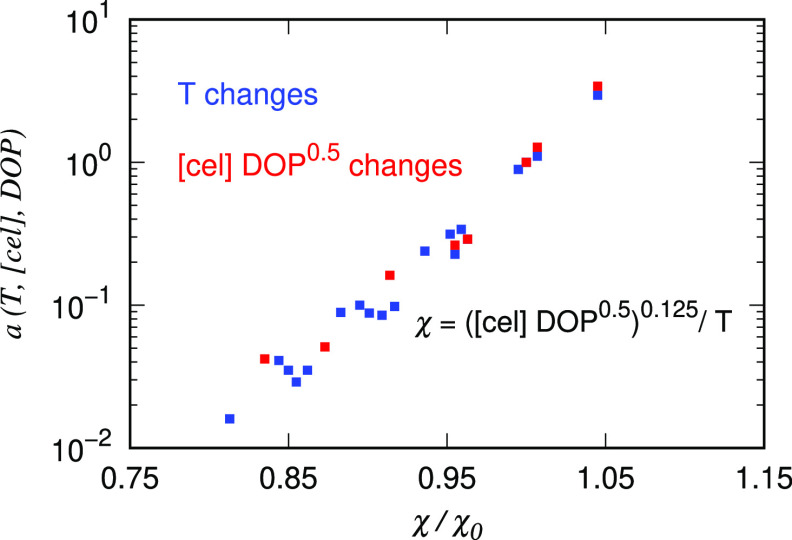
Horizontal shifting parameters obtained by the method of reduced variables collapsed according to χ ≡ ([cel]DOP^0.5^)^0.125^/*T* and normalized by the reference state χ_0_ at *T* = 25 °C, [cel] = 2%, and DOP = 2710.

The values of *a*(*T*, [cel], DOP) can be experimentally obtained by multiplying the shifting factors under the superposition laws of single variables. This calculation is based on the assumption of different superposition laws being independent (see the Supporting Information). Derivations starting from the Arrhenius relation,^65^ we attribute the variation of activation energy to the concentration and DOP. This argument has been justified by previous studies for a number of polymer solutions, including cellulose/IL systems.^46,88,93−95^ Explicitly, we assume the activation energy to follow a leading-order power-law relation with the overall size of the cellulose in the solution, which is scaled with [cel]DOP^ν^, where ν is the solvent quality factor. Finally, we can express *a*(*T*, [cel], DOP) in a general form as
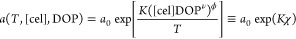
10where *a*_0_ is a normalization factor, and *K* and ϕ are the fitting parameters. The solvent quality factor was measured to be close to ν = 0.5, from which we found the best-fit exponent ϕ = 0.125 (see the Supporting Information), leading to a unified variable expressed in terms of the temperature, concentration, and DOP as χ ≡ ([cel]DOP^ν^)^0.125^/*T*. In Figure 5, we plot the values of *a*(*T*, [cel], DOP) against χ(*T*, [cel], DOP) normalized by a reference state χ_0_ at *T* = 25 °C, [cel] = 2% and DOP = 2710. All the shifting factors collapse onto a single curve.

**Figure 6:**
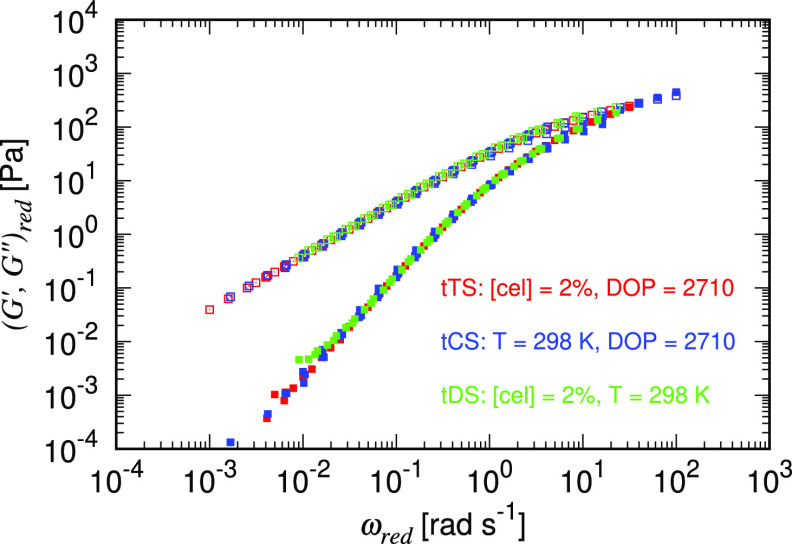
Super master curve including temperature (from Figure 3), concentration (from Figure 4), and DOP superposition by the generalized shifting parameters *a*(*T*, [cel], DOP) and *b*(*T*, [cel], DOP) letting (*G*′,*G*″)_red_ ≡ *b*(*T*,[cel],DOP)(*G*′,*G*″) and ω_red_ ≡ *a*(*T*, [cel], DOP) ω. The solid symbols indicate *G*′ and hollow symbols indicate *G*″.

**Figure 7:**
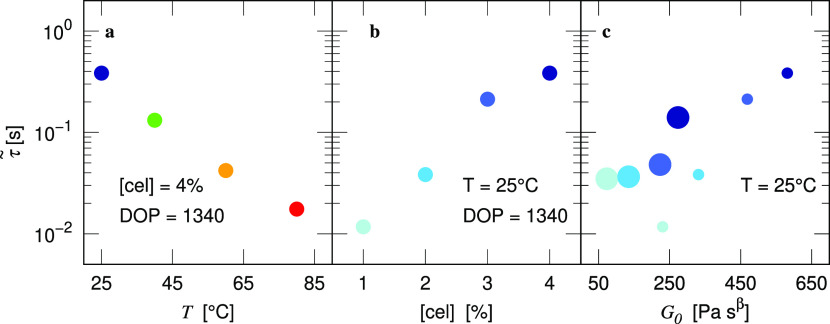
Characteristic relaxation time (τ̃) against (a) temperature (visualized by different marker colors) and (b) cellulose concentration (visualized by blue markers with different luminances) obtained from fitting to the FML model. (c) We show correlation of τ̃ and *G*_0_ for DOP = 1340 with [cel] = 1.0, 2.0, 3.0, 4.0% and DOP = 2710 with [cel] = 0.5, 1.0, 1.5, 2.0% [visualized by different marker sizes for DOP and luminances as in (b)].

We then utilize this general shifting factor *a*(*T*, [cel], DOP) from eq 10 to superpose all the curves of dynamic moduli measured under 25 different combinations of temperatures, concentrations, and DOP onto a “super master curve”, as shown in Figure 6. Not surprisingly, the overall trend can be successfully captured by the FML model. Asymptotic solutions of the FML model demonstrate that *G*′ ∼ ω^2−β^ and *G*″ ∼ ω at the low frequency end. At the high frequency end both *G*′ and *G*″ scale as ω^β^. A transition from viscous-like to solid-like behavior occurs at the crossover point ω_c_, at which *G*′ = *G*^″^, whose value can be determined using the fitting parameters from the FML model. However, the results should be carefully interpreted when the crossover point falls outside of the measured frequency because the extrapolation might fail to capture the trend of *G*′ and *G*″ faithfully and can lead to inaccurate results.^45^ To illustrate the dependence of the reptation relaxation time, which is defined as 1/ω_c_ and scales with τ̃ (see the Supporting Information), on temperature and concentration, we plot the extracted characteristic time τ̃ against different parameters in Figure 7. Figure 7a,b again shows opposing effects of the temperature (visualized by different marker hues) and concentration (visualized by different marker lightness) on the relaxation time of the solution. Furthermore, the correlation between elasticity, which increases with cellulose concentration or DOP (visualized by different marker sizes), and relaxation time is manifested in Figure 7c.

As introduced in Figure 2, the rheological profile of viscoelastic fluids measured by SAOS and steady shear measurements is remarkably similar but may sometimes fail for cellulose in IL at high shear rates due to nonaffine polymer deformation. To systematically probe this deviation at extremely high strains, we compared the steady shear rheology with extensional rheology and fitted both results using the Rolie-Poly model (Equation) to obtain a unified set of descriptive parameters.

**Figure 8:**
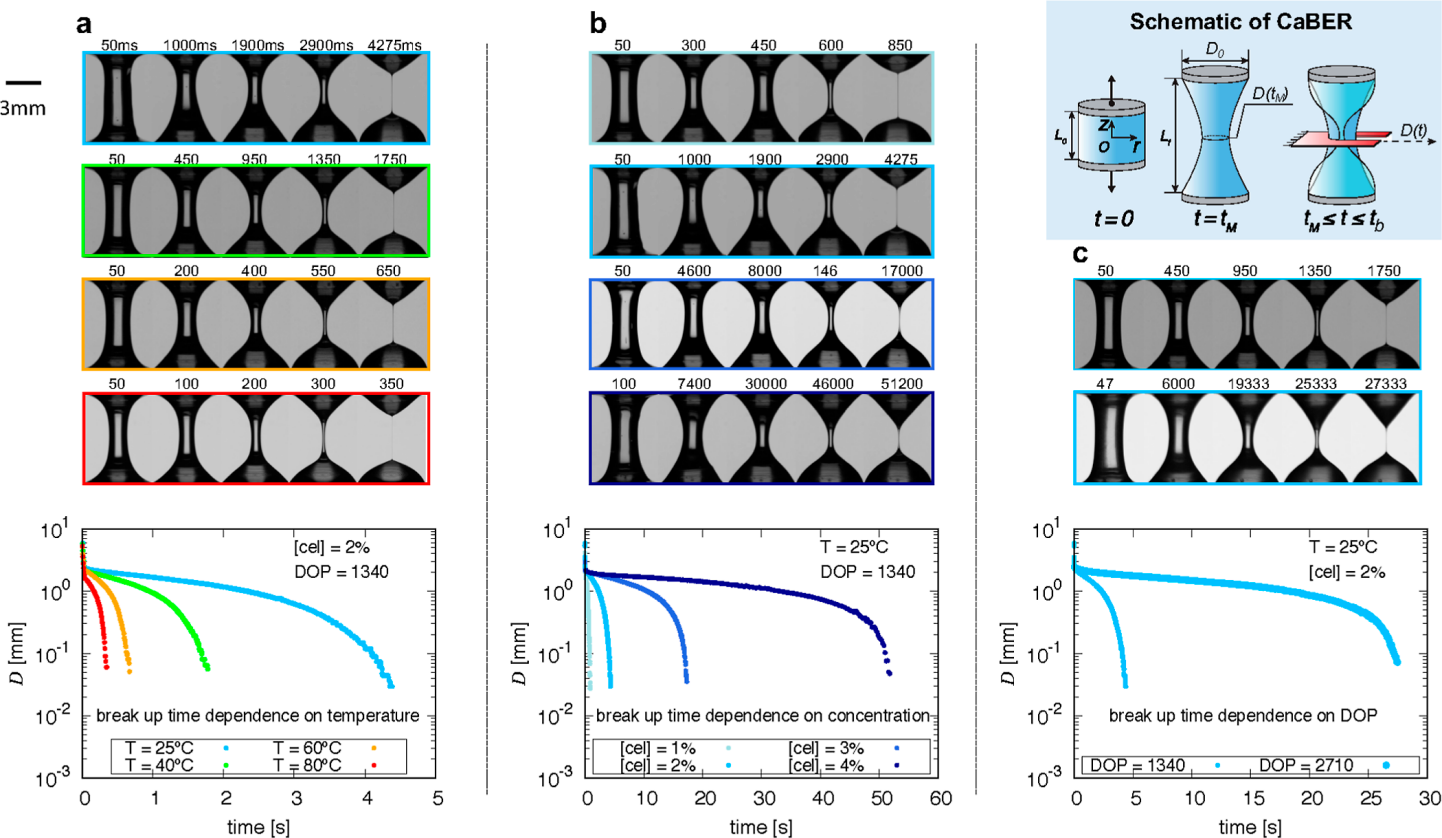
Snapshots and filament evolution of the capillarity-driven thinning profiles for the selected cellulose solutions. (a) 2% filter paper (DOP = 1340) solutions at 25, 40, 60, and 80 °C (blue, green, orange, and red, respectively). (b) Filter paper solutions at 25 °C with the concentrations of 1, 2, 3, and 4% (light to dark blue). (c) 2% Filter paper (DOP = 1340, small marker) and cotton fiber (DOP = 2710, large marker) solutions at 25 °C. Top-right corner: schematic of CaBER to illustrate the measurement process, where *t*_M_ = 40 ms is the designated motor actuation time and *t*_b_ is the filament breakup time.

Figure 8a–c shows the snapshots of the capillarity-driven thinning profiles for the cellulose solutions with indicated temperatures, concentrations, and DOP, respectively. These illustrate the temporal evolution of the minimal filament diameter *D*(*t*) as plotted in full time resolution below the corresponding snapshot sets. From the figures, the filament breakup time (where D → 0) increases as the temperature decreases, or as the concentration or DOP increases. The filament profiles exhibit curved shapes at the early stage of capillarity-driven thinning after the discs stop moving (i.e., *t* ≳ *t*_M_ = 40 ms) and become progressively cylindrical as the filament thinning progresses. This transition signifies different stress terms contributing to the capillarity-driven thinning dynamics at different stages of the filament thinning process.

**Figure 9:**
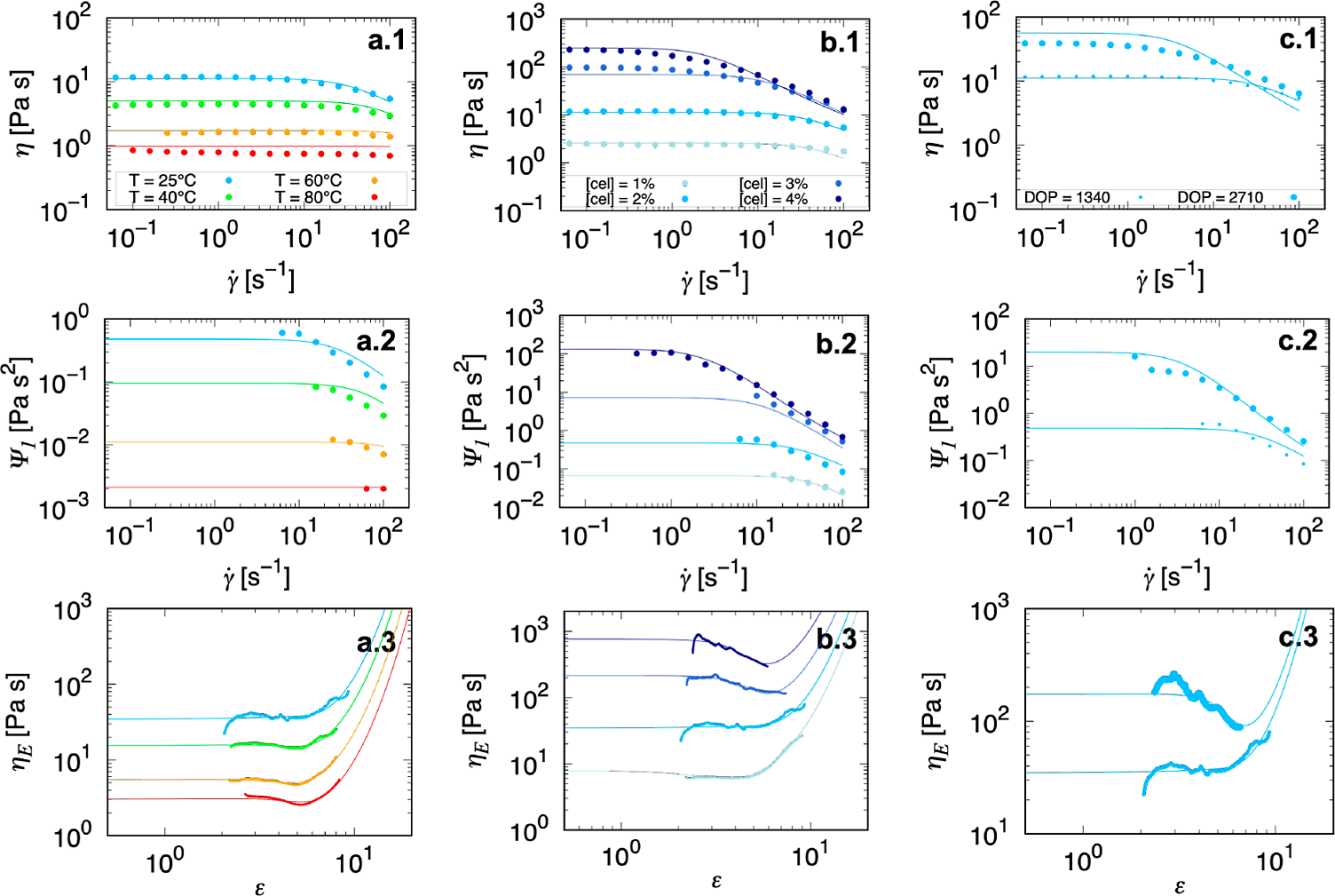
Shear viscosity (first row), first normal stress coefficient (second row), and apparent extensional viscosity (third row) for the two cellulose solutions fitted by the Rolie-Poly model (solid lines). (a1–3) 2% Filter paper (DOP = 1340) solutions at 25 °C (blue), 40 °C (green), 60 °C (orange), and 80 °C (red). (b1–3) Filter paper solutions at 25 °C with the concentrations of 1, 2, 3, and 4% (light to dark blue). (c1–3) 2% Filter paper (small marker) and cotton fiber (DOP = 2710, large marker) solutions at 25 °C.

We further extract the extensional viscosity from eq 8 as η = 2(2*X* – 1)Γ/(*D*ε̇) = Γ/[−*Ḋ*(*t*)], where *X* is the geometric correction factor quantifying the deviation of a filament profile from being cylindrical. In this study, the capillarity-driven thinning process is primarily dominated by the visco-capillary interaction because the Ohnesorge number is much larger than unity (calculated from the shear viscosity). As a result, we can approximate *X* = 0.7127 from the result for Newtonian fluids^83^ to simplify the calculation. Admittedly, the value of *X* may slightly deviate from this designated constant due to additional stress contributions from the cellulose conformation. However, the overall viscosity trend, primarily governed by the temporal evolution of *Ḋ*(*t*), is still retained. To show the transient process of material hardening in the extensional flow, we plot the extensional viscosity against the accumulated Hencky strain, which can be calculated by integrating the strain rate from *t* = 0 as , where *D*_0_ = 6 mm is the disk diameter and the initial filament diameter. The results from extensional rheology are reported with the shear data in Figure 9 following the same set of temperature, concentration, and DOP as in Figure 8.

As introduced above, the rationale for applying the Rolie-Poly model to fit experimental data is to obtain a set of constitutive parameters with a basis in molecular deformation that can be applied to both shear and extensional deformation. We comprehensively fitted the shear viscosity and first normal stress coefficient against the shear rate, and the extensional viscosity against the Hencky strain under an identical parameter set of zero-shear viscosity η_0_, disengagement time τ_d_, and Rouse time τ_s_. In the fitting of extensional data, an additional fitting parameter ε_0_ is added to the expression of the Hencky strain to account for a residue strain due to material loading before the material is stretched. In shear flow, data are replotted from Figure 2 with the new model fitted in Figure 9a1–c1. Shear stress data below the noise floor of the rheometer (10 μNm) was removed before fitting. In addition, the first normal stress coefficient Ψ_1_ is shown in Figure 9a2–c2 to give robustness and greater parameter certainty to the model fit. Normal force data below the noise floor of the rheometer (10 mN) were removed before fitting. From Figure 9a3–c3, the extensional viscosity is shown. The material under extension broadly experiences strain-softening behavior followed by a strain-hardening trend as the strain grows larger in an extensional flow, in contrast with the simple rate-thinning behavior in shear flows of the same material. This nonmonotonic trend has been reported for polymer solutions in the entanglement regimes^60^ but has never been accessed for dilute or semidilute cellulose spinning dopes.

As Figure 9 shows, the Rolie-Poly model provides a comprehensive fit to the zero-rate viscosities in both shear and extensional flows, and the transition to shear thinning or strain hardening over our range of concentrations and temperatures by both the disengagement time τ_d_ and the Rouse time τ_s_ for the cellulose solutions studied here. It is worth noting that the model recovers the nonmonotonic trend in the extensional flow at low temperatures and high concentrations or DOP. Based on insights from the model, the transient strain-softening behavior can be rationalized by the reorientation of the statistical polymer tubes toward the extensional direction, in which the mobility of polymer chains is increased.

**Figure 10:**
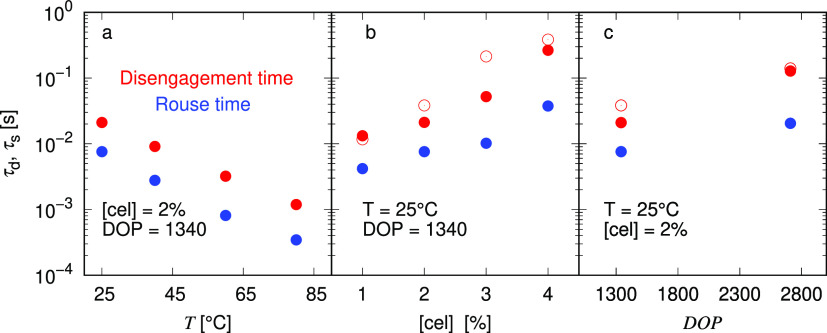
Disengagement time τ_d_ (red circles) and Rouse time τ_s_ (blue circles) obtained from fitting the Rolie-Poly model to both shear and extensional rheological measurements at different (a) temperatures, (b) concentrations, and (c) DOP as arranged in Figure 9. Open circles in (b) and (c) indicate the characteristic relaxation time τ̃ from Figure 7.

We then compare the fitting parameters separately against the temperature, concentration, and DOP as shown in Figure 10. Although these parameters are similar to those in the Cross^50,53,96^ and Carreau^45^ models used frequently with cellulose/IL solutions, the Rolie-Poly model is a constitutive model expressed in tensor forms with specific and, importantly, physically based parameter definitions. The fitted parameters can thus be readily extended to arbitrary three-dimensional flows, and the parameters can be further connected to the structural configuration from a microscopic perspective.

We explicitly plot the fitted parameters against the temperature, concentration, and DOP as shown in Figure 10. The zero-rate viscosity η_0_ increases with increasing concentration and decreases with increasing temperature, similar to the behavior of *G*_0_ and τ̃ from the FML model in Figure 7. Both disengagement and Rouse times increase as temperature decreases or concentration increases. When the DOP increases, the Rouse time τ_s_ increases more slowly than the disengagement time τ_d_. This is consistent with the scaling laws of both timescales with the number of entanglements per chain.^66^ Studies of the tube model have shown the onset of shear-thinning behavior at a critical shear rate of 1/*τ*_d_,^79^ which is attributed to the deformation of the entangled network. As a result, the disengagement time is expected to be close to the relaxation time τ̃ extracted from the LVE FML model, which is supported in this study by comparing with Figure 7.

Retrospectively, as shown in Figure 9, although the Rolie-Poly model provides a full-dimensional rheological framework, it is difficult to accurately extract all the model parameters from a single flow curve in which some parameters may be coupled or are less important in shaping the flow curve. Specifically, the discernible features exhibited in the shear flow for the Rolie-Poly model are primarily dominated by the tube reorientation, which corresponds to the disengagement time τ_d_, whereas the primary behavior exhibited in extensional flow at high strain rates is chain stretch, which is governed by the polymer conformation between entanglements, characterized by the Rouse time τ_s_. A full model fit requires reasonable certainty of both parameters. Therefore, it is necessary to apply both shear and extensional rheology for a comprehensive characterization to obtain accurate and physically interpretable fitting parameters.

### Perspective

Compared with previous studies,^48,71^ the application of the tube model for the cellulose/IL solutions presented in this study provides a more comprehensive rheological fitting by combining both shear and extensional data. The transient strain softening during the spinning process, which cannot be described by a simpler constitutive model, leads to a critical strain range where the spun fibers may experience an unbounded necking instability which undermines spinning performance. In fact, a qualitatively described “telescoping” failure has been observed as the main limitation in the spinning of cellulose fibers from cellulose in [C_2_C_1_Im][OAc] at high draw ratios.^53^ In our study, the application of the tube model not only provides a more accurate description of the mechanical behavior of a series of real spinning materials but can also promote a new understanding of the connection between fiber “spinnability” and the microscopic cellulose structures, dynamics, and interactions.

## Conclusions

In this study, we present a detailed rheological characterization of two celluloses with different DOP, namely cotton fibers and filter paper dissolved in 3-ethyl-1-methylimidazolium ([C_2_C_1_Im][OAc]). The linear viscoelasticity is probed by the SAOS. The results can be fitted into the FML model, and the Cox–Merz rule is found to apply within the measurement range. By analyzing the trend of the experimental data, we propose a new scheme of master superposition that incorporates the effects of the temperature, concentration, and DOP with a new combined variable system. Furthermore, the non-LVE rheological behavior at high strain rates is probed in both shear and extensional flows using steady-state shear tests and CaBER. A well-established tube model, the Rolie-Poly model, is applied to provide a comprehensive fitting framework regardless of the flow type. The obtained, physically interpretable model parameters show informative trends based on varied solution test temperatures, concentrations, and DOP and are consistent with the scheme of superposition used for data on linear viscoelasticity. This study presents a systematic characterization method for concentrated cellulose/IL solutions, and the results are closely connected to the material deformation in a fiber spinning process. We hope for potential applications of this study to support the optimization of designing and modeling of a more efficient industrial process by allowing sensible characterization and design of cellulose in IL solvents.

## Supporting Information Available

Solution preparation, intrinsic viscosity estimates, and radius of gyration, analytical solutions and asymptotes predicted by the FML model, shear viscosity of [C_2_C_1_Im][OAc], additional rheological measurements of cellulose/[C_2_C_1_Im][OAc] solutions that follow the Cox–Merz rule, tTS for filter paper at 4%, derivation of the generalized shifting parameters, and variation of solvent quality with temperature (PDF)
