# Supplementary material for: Understanding the Dynamics of Cellulose Dissolved in an Ionic Liquid Solvent Under Shear and Extensional Flows
Source: Biomacromolecules. 2022 Apr 20;23(5):1958–69. doi: 10.1021/acs.biomac.1c01623 (PMC9707809; doi:10.1021/acs.biomac.1c01623)
Supplement: Supplementary file 1 — bm1c01623_si_001.pdf [file bm1c01623_si_001.pdf]

Supporting Information: Understanding the dynamics of cellulose dissolved in an ionic liquid solvent under shear and extensional flows

Crystal E. Owens¶,† Jianyi Du¶,† and Pablo B. Sánchez*,†,‡

†*Hatsopoulos Microfluids Laboratory, Department of Mechanical Engineering, Massachusetts Institute of Technology, Cambridge, MA 02139, United States*

‡*University of Vigo, Vigo, Spain*

¶*These authors contributed equally.*

E-mail: pabsanchez@uvigo.es

Solution preparation, intrinsic viscosity estimates, and radius of gyration

By extrapolating curves of zero-shear viscosity, η_0 vs concentration, [cel], for the data shown in Figure 2, we have calculated the intrinsic viscosity of our cellulose solutions, $[\eta]$. The intrinsic viscosities are found to be temperature-independent with mean values of 2.97 dL/g for 2710 DOP (439 kDa) and 1.34 dL/g for 1340 DOP (217 kDa), giving a relationship of $MW = 0.0066[\eta]$ for molecular weight in kg/mol and $[\eta]$ in dL/g. Following the equations in ASTM D4243-99 (specifically, assuming a Mark-Houwink plot with exponent 1) and comparing with prior results of intrinsic viscosity for cellulose/$[C_2C_1Im][OAc]$,[1] these results may suggest that our cellulose dissolution process has depolymerized the cellulose to 70-90% of its original

length, which has been shown for cel/IL at similar cellulose concentrations and DOP for similar lengths of time and temperature.[1–5] Further, the temperature-independence of $[\eta]$ indicates lack of substantial contribution of interfacial effects of cellulose, which is known to form a "skin" on the surface of ionic liquid solutions and which, when present, disturbs rheological measurements in dilute concentration regimes and in testing with rheometer fixtures that use a large interface open to air.[1]

Using this fitted intrinsic viscosity and the Fox-Flory relationship,[6] we estimate the radius of gyration of our cellulose to be 16 nm to 31 nm for our high-DOP material and 10 nm to 19 nm for our low-DOP material, similar to those measured by scattering methods on cellulose of similar DOP in other ionic liquids.[6]

Analytical solutions and asymptotes predicted by the Fractional Maxwell Liquid model

The dynamic moduli predicted by the Fractional Maxwell Liquid model can be analytically expressed as a function of the oscillatory frequency, as shown in Figure S1. Power-law trendlines arise at the extremity of frequency for both G' and G''. From asymptotic analysis, all the power-law asymptotes can be expressed in Table S1, in which $\widetilde{\tau} = (\mathbb{V}/\mathbb{G})^{1/(1-\beta)}$ is a characteristic timescale as defined in text surrounding Equation 6 in the manuscript.

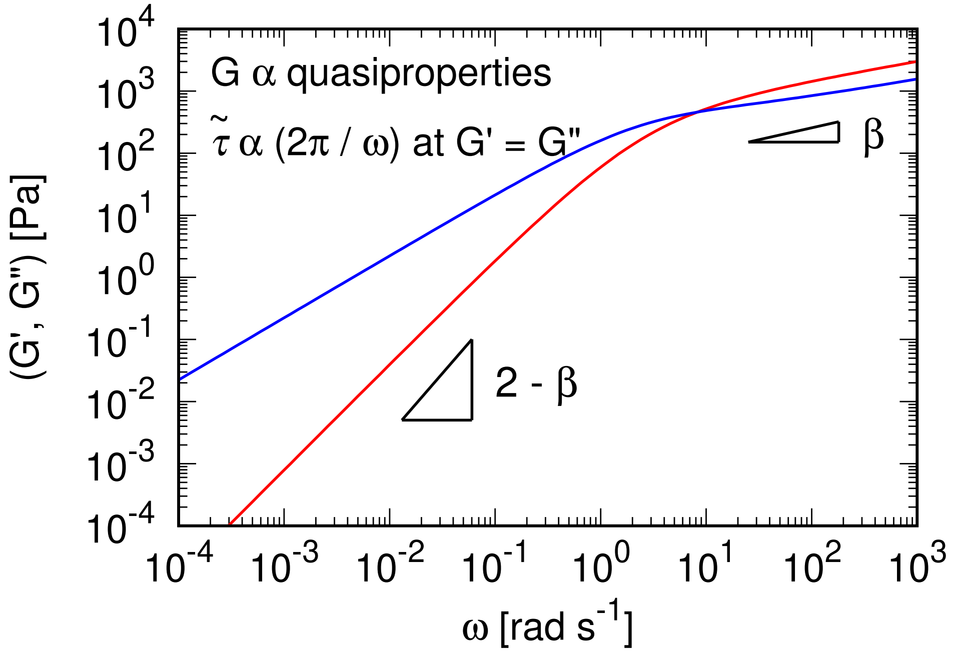

Figure S1: Dynamic moduli against oscillatory frequency predicted by the FML and asymptotic power-laws at both extremities of frequency. Red and blue lines correspond to the storage and loss moduli, respectively.

From the analytical expressions in Equation 6 of the manuscript, a crossover frequency at which $G' = G''$ can be expressed as

$$\omega_c = \frac{\left[\sqrt{2}\cos\left(\frac{\pi\beta}{2} + \frac{\pi}{4}\right)\right]^{1/(\beta-1)}}{\widetilde{\tau}}. \tag{1}$$

It is clear that when $\beta = 0$, $\omega_c = 1/\widetilde{\tau}$, which reduces to the classic result from the Maxwell model.[7]

Table S1: Asymptotic power-law solutions at the extremity of frequency for both storage and loss moduli.

Limit of frequency	G'	G''
$\omega\widetilde{\tau} \ll 1$	$\sim \omega^{2-\beta}$	$\sim \omega$
$\omega\widetilde{\tau} \gg 1$	$\sim \omega^{\beta}$	$\sim \omega^{\beta}$

For reference, the fitting parameters from the FML model for cellulose/IL solutions at

varying concentrations and DOPs are summarized in Table S2.

Table S2: Constitutive parameters fitted from the FML model for both cellulose sources at different concentrations.

Material	[cel] (%)	τ (ms)	β	$\mathbb{G}$ (Pa s$^\beta$)
Cotton fibers (DOP=2710)	0.5	0.0349	0.389	73.2
	1.0	0.0365	0.304	134.5
	1.5	0.0481	0.345	222.9
	2.0	0.1402	0.315	273.6
Filter paper (DOP=1340)	1.0	0.0117	0.316	230.5
	2.0	0.0384	0.316	331.5
	3.0	0.2130	0.361	469.0
	4.0	0.3850	0.297	581.5

Shear viscosity of $[C_2C_1Im][OAc]$

To characterize the shear rheology of ionic liquid (IL) solvents under different thermal conditions, the stock solvent 1-ethyl-3-methylimidazolium acetate $[C_2C_1Im][OAc]$ was measured on a commercial rolling-ball viscometer (Lovis 2000M/ME, Anton Paar) over a temperature range from 290 K to 350 K. As Figure S2 shows, the shear viscosity η increases dramatically as the temperature decreases. Here, two semi-empirical relations are applied to interpret the viscosity measurements: namely, the Arrhenius relation and the Vogel-Fulcher-Tammann (VFT) relation.[7] They can be expressed as

$$\text{Arrhenius: } \frac{\eta(T)}{\eta(T_0)} = \exp\left[\frac{\Delta H}{R_g}\left(\frac{1}{T} - \frac{1}{T_0}\right)\right], \tag{2a}$$

$$\text{VFT: } \frac{\eta(T)}{\eta(T_0)} = \exp\left(\frac{B}{T - T_{VF}}\right), \tag{2b}$$

where T_0 is a reference temperature. In the Arrhenius relation, ΔH and R_g correspond to the activation energy and the gas constant, respectively. In the VFT relation, T_{VF} and B are fitting parameters. Both relations in Equation 2 exhibit strong temperature dependent when

T is close to T_0 or T_{VF}. As Figure S2 shows, both fitting lines agree with the experimental data in the temperature range of interest.

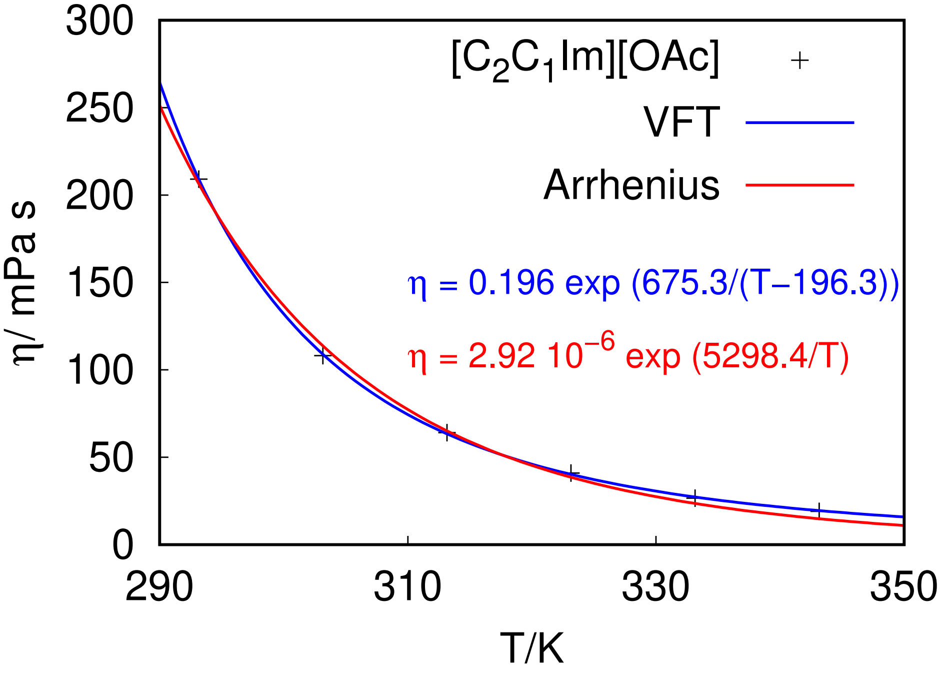

Figure S2: Viscosity against temperature of $[C_2C_1Im][OAc]$ and fitting lines to the Vogel-Fulcher-Tammann (blue) and Arrhenius (red) relations.

Additional rheological measurements of cellulose/$[C_2C_1Im][OAc]$ solutions that follow the Cox-Merz rule

Additional experiments were performed on the measured cellulose/IL solutions to validate the empirical Cox-Merz rule particularly at high concentration and DOP. As Figure S3 shows, the steady shear (filled symbols) and complex (hollow symbols) viscosities of cellulose/IL solutions agree over varying temperatures, cellulose concentrations, and DOPs. Under all conditions, both viscosities coincide over the measured range of shear rates ($0.1\,s^{-1}$ to $100\,s^{-1}$), which justify the generality of the Cox-Merz rule over a variation of material and environmental conditions for our material system.

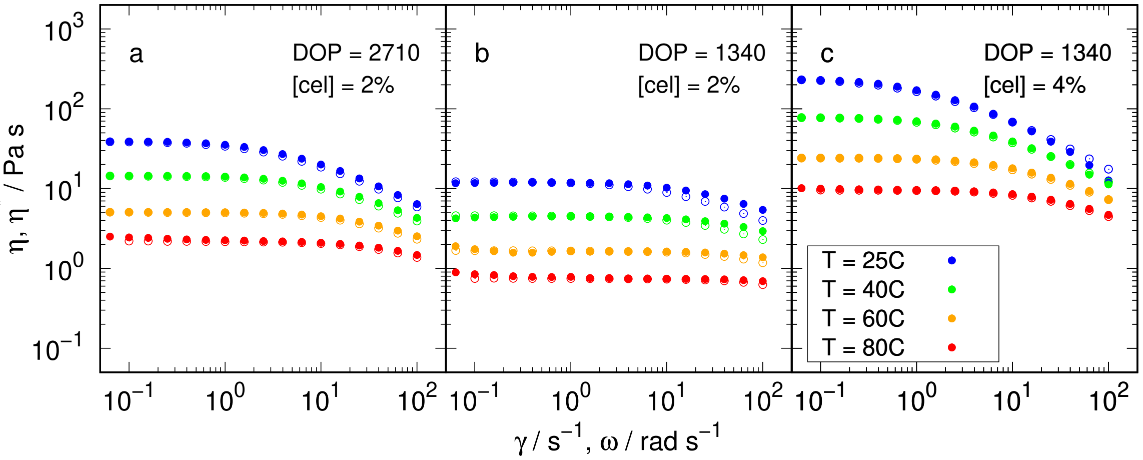

Figure S3: Steady-shear (filled symbols) and complex (hollow symbols) viscosity of cellulose/[C_2C_1Im][OAc] solutions against shear rate and frequency at different temperature, concentration and degree of polymerization (DOP).

Time-temperature superposition for filter paper at 4%

The dynamic moduli for IL solutions of filter paper (DOP=1340) are also measured and a time-temperature superposition is performed as shown in Figure S4.

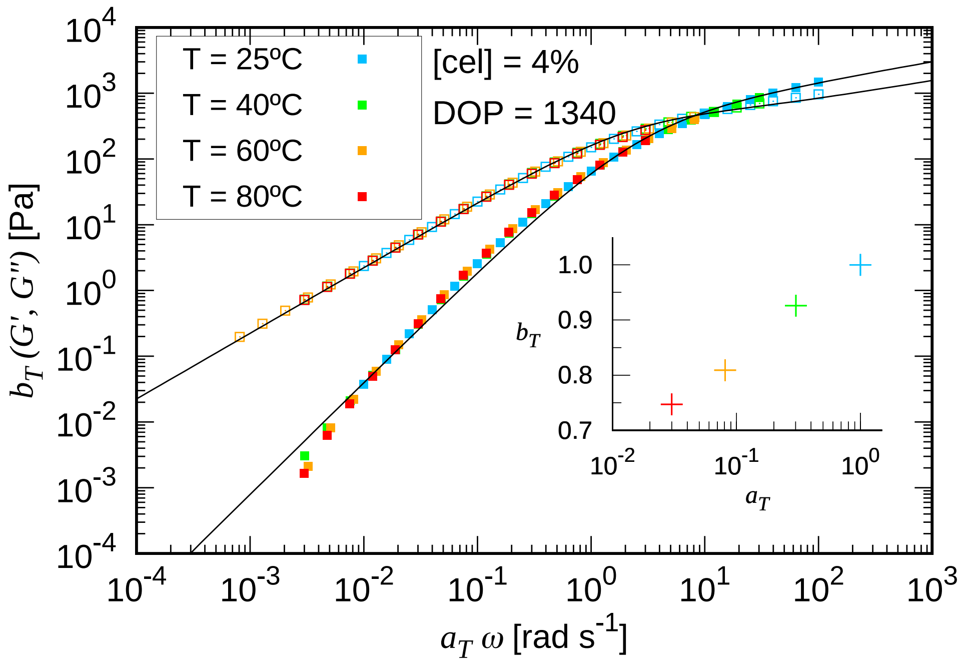

Figure S4: Superposed dynamic moduli for filter paper dissolved in $[C_2C_1Im][OAc]$ at 4% at varying temperature with horizontal and vertical shifting. Solid black lines account for the fitting of the collapsed data to FML model. Shift factors a_T and b_T are shown in the inset of the figure.

Derivation of the generalized shifting parameters

In this paper, a generalized shifting factor $a(T, [\mathrm{cel}], \mathrm{DOP})$ is applied to collapse the linear viscoelastic responses under different environmental and material conditions onto a single mastercurve. This multivariate superposition can be applied to predict the responses of the cellulose/IL system under a wide range of conditions. In addition, the validity of such superposition reveals cooperative and competitive collective impacts from varied external conditions and material structures on the rheology of this system. To better justify the explicit expression of Equation 10 in the manuscript, the activation energy E_a for each combination of solutions is extracted from fitting to the Arrhenius relation, as shown in Figure S5a and b. It is evident that the activation energy at varying concentrations and DOPs are distinct. In addition, a solid line in Figure S5b shows the activation energy measured by ^{1}H NMR,[8] which

was measured as a function of concentration of microcrystalline cellulose in $[C_2C_1Im][OAc]$ (DOP ≈180). Their results agree quantitatively with our collapsed-system plot. This correspondence indicates that the concentration dependence of activation energy observed here by rheology can be broadly attributed to the variation in activation energy between free ions (both anions and cations) and ions associated with cellulose to a greater proportion as cellulose concentration increases, which hinders diffusion of the ions.[8]

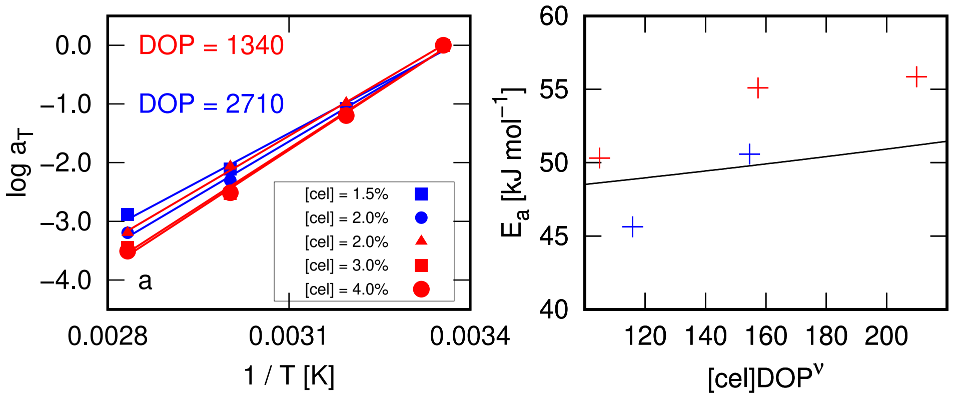

Figure S5: (a) Shifting factors obtained by tTS and fittings calculated as log a_T versus $1/T$. (b) Activation energy for flow as a function of [cel]DOP$^\nu$ determined from the slopes in (a). The solid black line indicates activation energy measured by ^{1}H NMR with changing cellulose concentration from Ries et al.[8]

If an explicit expression can be obtained for the activation energy as a function of concentration and DOP, the Arrhenius relation can be readily generalized to produce a superposition law for all three parameters. Here, a variable of [cel]DOP$^\nu$ is used, which combines the contributions from both concentration and DOP. From the perspective of polymer physics, this variable scales with the summed end-to-end distance for all polymer chains per volume. As shown in Figure S5b, by plotting the activation energy against this variable, a collapsed power-law trend can be identified, and the power exponent can be fitted as approximately 0.125. Consequently, to compare the shifting effects under different conditions using a unified framework, while keeping the Arrhenius relation valid, we assume a generalized shifting

factor to follow

$$a(T, [\text{cel}], \text{DOP}) = C \exp\left[K \frac{([\text{cel}]\text{DOP}^{\nu})^{0.125}}{T}\right], \tag{3}$$

which coincides with the definition of Equation 10 in the manuscript. The value of C is normalized so that $a = 1$ at a selected reference state (*viz.*, 2% cotton fiber/IL solutions at $T = 298.15\,\text{K}$ in this paper). Numerically, this generalized shifting factor equals to the multiplication of the shifting factors for individual parameters. Due to commutative property, this corresponds to a path-independent shifting, *i.e.*,

$$\begin{aligned}
&a_T(T_2; T_1, [\text{cel}]_1, \text{DOP}_1) a_C([\text{cel}]_2; T_2, [\text{cel}]_1, \text{DOP}_1) a_D(\text{DOP}_2; T_2, [\text{cel}]_2, \text{DOP}_1)\\
&= a_C([\text{cel}]_2; T_1, [\text{cel}]_1, \text{DOP}_1) a_T(T_2; T_1, [\text{cel}]_2, \text{DOP}_1) a_D(\text{DOP}_2; T_2, [\text{cel}]_2, \text{DOP}_1)\\
&= \ldots\\
&= \frac{a(T_2, [\text{cel}]_2, \text{DOP}_2)}{a(T_1, [\text{cel}]_1, \text{DOP}_1)},
\end{aligned} \tag{4}$$

where T_1, T_2, $[\text{cel}]_1$, $[\text{cel}]_2$, DOP_1 and DOP_2 are arbitrarily chosen within the allowed range and the notation $a_x(x_2; T_1, [\text{cel}]_1, \text{DOP}_1)$ indicates the shifting along variable-axis x to the value of x_2 from a reference state $(T_1, [\text{cel}]_1, \text{DOP}_1)$. a_T, a_C and a_D correspond to the shifting parameters for temperature, concentration and DOP, respectively. In Equation 4, expressions on different lines visualize distinct paths of shifting from state $(T_1, [\text{cel}]_1, \text{DOP}_1)$ to $(T_2, [\text{cel}]_2, \text{DOP}_2)$, which equal to the ratio between the generalized shifting factors of the destination and initial states.

Here, an example is provided in Figure S6 to show the path independence between cellulose concentration and DOP by shifting from state (25 °C, 1%, 1340) to (25 °C, 2%, 2710) along two distinct paths through (A) (25 °C, 2%, 1340), and (B) (25 °C, 1%, 2710). Since the temperature is fixed at 25 °C, the shifting factors between two adjacent states (a_C and a_D) can be readily determined by overlapping the dynamic moduli through horizontal shifting, as shown in each subfigure, where the target dynamic moduli in each step are marked as semi-

transparent. These four shifting factors can be obtained as $a_C(2\%; 25\,°\text{C}, 1\%, 1340) = 0.195$, $a_D(2710; 25\,°\text{C}, 2\%, 1340) = 0.35$, $a_D(2710; 25\,°\text{C}, 1\%, 1340) = 0.43$ and $a_C(2\%; 25\,°\text{C}, 1\%, 2710) = 0.162$. As a result, the overall shifting factors along path A and B can be calculated as

$$\begin{aligned} a_C(2\%; 25\,°\text{C}, 1\%, 1340)a_D(2710; 25\,°\text{C}, 2\%, 1340) = 0.0683 \\ a_D(2710; 25\,°\text{C}, 1\%, 1340)a_C(2\%; 25\,°\text{C}, 1\%, 2710) = 0.0697, \end{aligned} \tag{5}$$

which are reasonably close to each other. Such path independence can be readily justified from the independence of variables, and Equation 4 provides a viable expression for the calculation of the generalized shifting factor.

Variation of solvent quality with temperature

In a plot of the specific viscosity, $\eta_{sp} \equiv (\eta_0 - \eta_s)/\eta_s$, versus concentration, the power-law behavior of the concentration dependence is indicative of the solvent quality, *i.e.*,

$$\eta_{sp}([\text{cel}]) \propto [\text{cel}]^{3/(3\nu-1)} \text{ for } [\text{cel}] > [\text{cel}]_e, \tag{6}$$

It has been reported before that solvent quality of cellulose in $[C_2C_1Im][OAc]$ is approximately a theta solvent at room temperature.[9,10] Our measurements agree, as shown in Figure S7, that $\nu \approx 0.55$ at $T = 25\,°\text{C}$, and further show increasing solvent quality from 0.55 to 0.58 as the temperature increases from 20 °C to 80 °C.

References

(1) Utomo, N. W.; Nazari, B.; Parisi, D.; Colby, R. H. Determination of Intrinsic Viscosity of Native Cellulose Solutions in Ionic Liquids. *J. Rheol.* **2020**, *64*, 1063–1073.

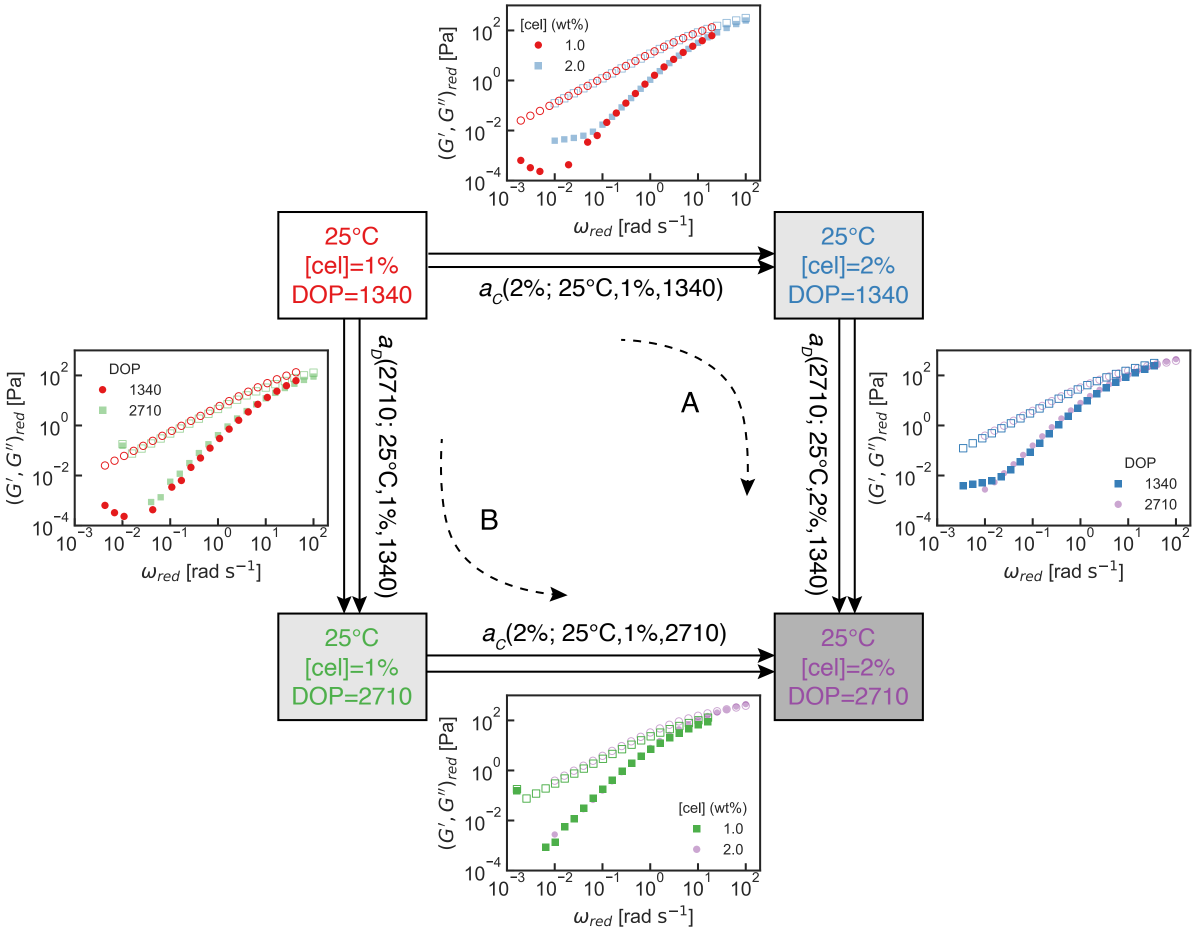

Figure S6: Two paths of shifting from the initial state $T_1 = 25\,°\text{C}$, $[\text{cel}]_1 = 1\%$ and $\text{DOP}_1 = 1340$ (shaded in white) to the destination state $T_1 = 25\,°\text{C}$, $[\text{cel}]_2 = 2\%$ and $\text{DOP} = 2710$ (shaded in dark grey): (A) through $T_1 = 25\,°\text{C}$, $[\text{cel}]_2 = 2\%$ and $\text{DOP}_1 = 1340$; (B) through $T_1 = 25\,°\text{C}$, $[\text{cel}]_1 = 1\%$ and $\text{DOP}_2 = 2710$ (both shaded in light grey). In each step, shifting factors of either a_C or a_D can be determined from the experimental data, and the superposed dynamic moduli in each step are plotted on the side.

(2) Utomo, N. W. Rheology of Native Cellulose Solutions in Ionic Liquids. M.Sc. thesis, The Pennsylvania State University, 2019.

(3) De Silva, R.; Vongsanga, K.; Wang, X.; Byrne, N. Cellulose Regeneration in Ionic Liquids: Factors Controlling the Degree of Polymerisation. *Cellulose* **2015**, *22*, 2845–2849.

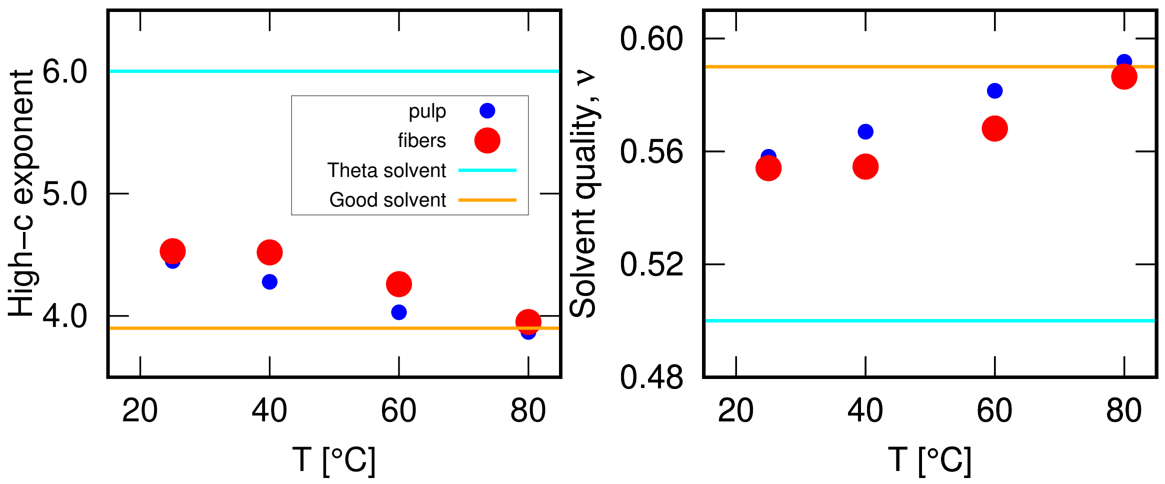

Figure S7: Change of high-concentration exponent and solvent quality for different temperatures indicating (a) the high-concentration exponent and (b) the calculated solvent quality ν.

(4) Michud, A.; Hummel, M.; Haward, S.; Sixta, H. Monitoring of Cellulose Depolymerization in 1-Ethyl-3-Methylimidazolium Acetate by Shear and Elongational Rheology. *Carbohydr. Polym.* **2015**, *117*, 355–363.

(5) Koide, M.; Wataoka, I.; Urakawa, H.; Kajiwara, K.; Henniges, U.; Rosenau, T. Intrinsic Characteristics of Cellulose Dissolved in an Ionic Liquid: The Shape of a Single Cellulose Molecule in Solution. *Cellulose* **2019**, *26*, 2233–2242.

(6) Gubitosi, M.; Duarte, H.; Gentile, L.; Olsson, U.; Medronho, B. On Cellulose Dissolution and Aggregation in Aqueous Tetrabutylammonium Hydroxide. *Biomacromolecules* **2016**, *17*, 2873–2881.

(7) Bird, R. B.; Armstrong, R. C.; Hassager, O. *Dynamics of Polymeric Liquids*, 2nd ed.; Wiley: New York, 1987.

(8) Ries, M. E.; Radhi, A.; Keating, A. S.; Parker, O.; Budtova, T. Diffusion of 1-Ethyl-3-methyl-imidazolium Acetate in Glucose, Cellobiose, and Cellulose Solutions. *Biomacromolecules* **2014**, *15*, 609–617.

(9) Haward, S. J.; Sharma, V.; Butts, C. P.; McKinley, G. H.; Rahatekar, S. S. Shear and Extensional Rheology of Cellulose/Ionic Liquid Solutions. *Biomacromolecules* **2012**, *13*, 1688–1699.

(10) Gericke, M.; Schlufter, K.; Liebert, T.; Heinze, T.; Budtova, T. Rheological Properties of Cellulose / Ionic Liquid Solutions : From Dilute to Concentrated States. *Biomacromolecules* **2009**, *10*, 1188–1194.
